# Genetic encoding of an esophageal motor circuit

**DOI:** 10.1016/j.celrep.2022.110962

**Published:** 2022-06-14

**Authors:** Tatiana C. Coverdell, Ruei-Jen Abraham-Fan, Chen Wu, Stephen B.G. Abbott, John N. Campbell

**Affiliations:** 1 Biomedical Sciences Graduate Program, University of Virginia, Charlottesville, VA 22903, USA; 2 Department of Biology, University of Virginia, Charlottesville, VA 22903, USA; 3 Beth Israel Deaconess Medical Center, Boston, MA 02215, USA; 4 Department of Pharmacology, University of Virginia, Charlottesville, VA 22903, USA; 5 These authors contributed equally; 6 Lead contact

## Abstract

Motor control of the striated esophagus originates in the nucleus ambiguus (nAmb), a vagal motor nucleus that also contains upper airway motor neurons and parasympathetic preganglionic neurons for the heart and lungs. We disambiguate nAmb neurons based on their genome-wide expression profiles, efferent circuitry, and ability to control esophageal muscles. Our single-cell RNA sequencing analysis predicts three molecularly distinct nAmb neuron subtypes and annotates them by subtype-specific marker genes: *Crhr2, Vipr2*, and *Adcyap1*. Mapping the axon projections of the nAmb neuron subtypes reveals that Crhr2^nAmb^ neurons innervate the esophagus, raising the possibility that they control esophageal muscle function. Accordingly, focal optogenetic stimulation of cholinergic *Crhr2*^+^ fibers in the esophagus results in contractions. Activating Crhr2^nAmb^ neurons has no effect on heart rate, a key parasympathetic function of the nAmb, whereas activating all of the nAmb neurons robustly suppresses heart rate. Together, these results reveal a genetically defined circuit for motor control of the esophagus.

## In brief

Primary motor neurons for the esophagus reside in the nucleus ambiguus (nAmb) of the hindbrain, but little is known about their molecular identity. Coverdell et al. find that the nAmb comprises three molecularly and anatomically distinct neuron subtypes, one of which selectively innervates and can contract esophageal muscle.

## Graphical Abstract

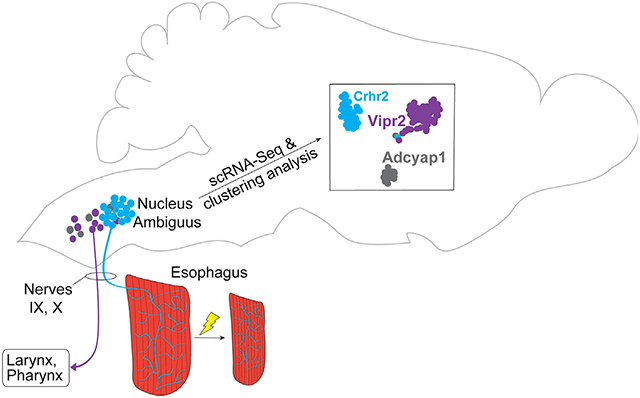

## INTRODUCTION

Primary motor neurons for the striated esophagus reside in the nucleus ambiguus (nAmb), an evolutionarily conserved vagal nucleus that also provides motor input to laryngeal and pharyngeal muscles (Bieger and Hopkins, 1987; Holstege et al., 1983; Lawn, 1966; Powley et al., 2013; Taylor et al., 1999, 2014). nAmb neurons innervating the esophagus, larynx, and pharynx, together known as special visceral efferent (SVE) neurons, are largely distinct in their anatomy and function (Bieger and Hopkins, 1987; Holstege et al., 1983; McGovern and Mazzone, 2010; Nunez-Abades et al., 1992). In addition to its motor neurons, the nAmb contains parasympathetic pre-ganglionic neurons, also known as general visceral efferent (GVE) neurons. These GVE neurons control smooth and cardiac muscles of the cardiorespiratory system (Mazzone and Canning, 2013; McAllen and Spyer, 1978; Nosaka et al., 1979). The cellular heterogeneity of the nAmb has made it difficult to isolate the esophageal motor neurons experimentally, limiting what is known about their molecular identity and organization.

Neuron subtypes can be classified based on differentially expressed genes, which can then be leveraged to access and genetically manipulate each subtype. We used this approach to identify nAmb neuron subtypes and generate hypotheses about their roles in esophageal control. Targeting each nAmb neuron subtype with intersectional genetic strategies, we then tested our hypotheses by (1) mapping the axon projections of the subtypes to the upper airways and esophagus and (2) optogenetically activating the subtypes *in vivo* to assess their control of esophageal muscle and heart rate. Our studies uncovered one molecular subtype of nAmb neurons that selectively innervates the esophagus. Activating these neurons caused esophageal contractions but did not affect heart rate. Together, our results suggest a genetic logic to the organization of the nAmb and identify its esophageal motor neurons by their gene expression, anatomy, and function.

## RESULTS

### Molecular identification of nAmb neuron subtypes

To identify nAmb neuron subtypes in mice, we profiled their genome-wide mRNA expression by single-nuclei RNA sequencing, sNuc-seq (Habib et al., 2016; Tao et al., 2021; Todd et al., 2020). To enrich for nAmb neurons in our analysis, we used a mouse line that expresses Cre recombinase down-stream of the endogenous *Chat* gene locus (Chat-Cre) (Rossi et al., 2011). We validated Cre activity in that mouse line by crossing it to a Cre reporter strain, H2b-TRAP (Roh et al., 2017), which fluorescently labels cells with Cre activity. We then labeled all peripherally projecting nAmb neurons using a systemically injected retrograde tracer, FluoroGold (FG; Chang et al., 2015; Cheng and Powley, 2000; Leong and Ling, 1990). Cre recombinase activity was present in 100% of FG-labeled nAmb neurons (396/396 neurons; n = 3 mice; Figure S1A), indicating that all peripherally projecting nAmb neurons have Chat-Cre activity. Furthermore, we used RNA fluorescence *in situ* hybridization (RNA FISH) to co-localize *Cre* mRNA with *Chat* mRNA in nAmb neurons of Chat-Cre mice and observed that all *Cre*^+^ cells were also *Chat*^+^ (103/103 of *Chat*^+^ neurons were *Cre*^+^; *Cre* mRNA was only detected in hindbrain cells that expressed *Chat*; n = 3; Figure S1B). Thus, our results indicate that the Chat-Cre mouse expresses Cre in all *Chat*^+^ and peripherally projecting nAmb neurons.

**Figure 1.**
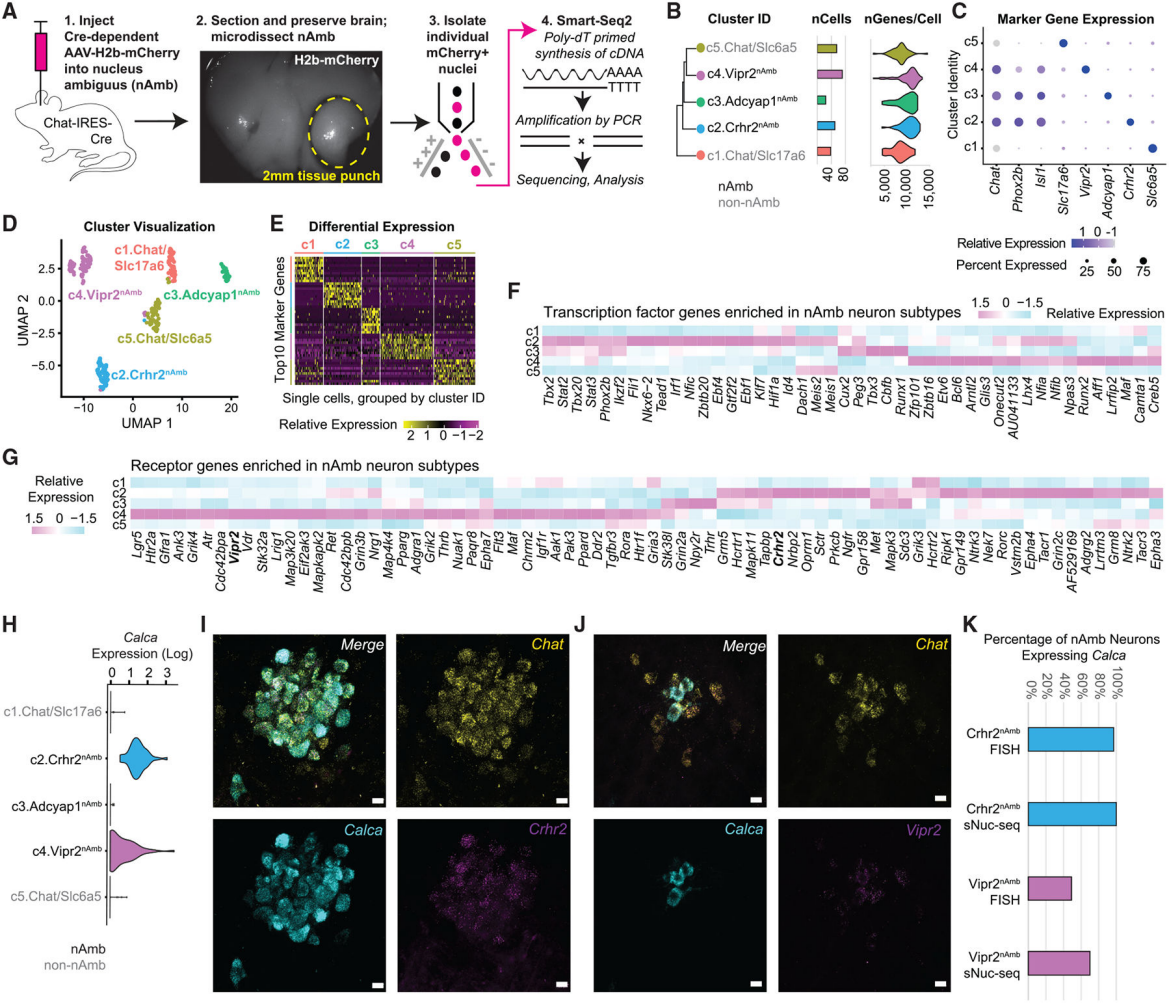
Molecular identification of nucleus ambiguus (nAmb) neuron subtypes (A) Schematic of single-nuclei RNA sequencing (sNuc-seq) workflow. (B) Relatedness of neuron clusters, number of cells per cluster, and number of genes detected per cell. (C) Dot plot of nAmb regional genes (*Chat, Phox2b, Isl1*) and cluster marker genes. (D) UMAP (uniform manifold approximation and projection) visualization of clustered data. (E) Single-cell expression heatmap of top marker genes for each cluster. (F) Cluster-level average expression of transcription factor genes. (G) Cluster-level average expression of receptor genes. (H) Violin plots of *Calca* expression. (I) Fluorescence *in situ* hybridization of *Calca*, *Chat*, and *Crhr2* mRNA in the “compact” (rostral) nAmb. Scale bar, 20 μm. (J) Fluorescence *in situ* hybridization of *Calca, Chat*, and *Vipr2* mRNA in the “semi-compact” (caudal) nAmb. Scale bar, 20 μm. (K) Percentage of *Calca*-expressing Crhr2^nAmb^ neurons and Vipr2^nAmb^ neurons in FISH and sNuc-seq studies. nAmb neurons identified in FISH by systemic FluoroGold labeling and in sNuc-seq by *Isl1* expression. The numbers of *Calca*^+^ cells as a fraction of the total cells in that group are shown in parentheses.

To fluorescently label nAmb neurons for sNuc-seq, we injected a Cre-dependent AAV vector, AAV-DIO-H2b-mCherry, into the ventrolateral medulla of Chat-Cre mice, then isolated single, mCherry^+^ cell nuclei from the nAmb area and sequenced their mRNA with Smart-seq2 to near-saturating depth for gene detection (Picelli et al., 2014; Tao et al., 2021; Todd et al., 2020; Figures 1A and S1C). Removing low-complexity transcriptomes (<2,000 genes per nucleus) left 238 single-nuclei transcriptomes for further analysis. Clustering these based on their expression of highly variable genes (Hao et al., 2021; Tao et al., 2021; Todd et al., 2020) yielded five cell clusters, which we annotated based on marker genes (Figures 1B-1D). These cell clusters differed substantially in their expression of transcription factors, receptors, and other genes (Figures 1E-1G and S1D).

We then compared cluster-level gene expression to identify the cell type represented by each cluster. Confirming their identity as cholinergic neurons, all of the clusters expressed the neuron marker genes *Tubb3* and *Syn1*, the cholinergic marker gene *Chat*, but few to no glial marker genes (Figure S1E). However, only three of the five clusters expressed the nAmb marker genes *Isl1* and *Phox2b* (Figures 1C and S1F), supporting their identity as nAmb neurons. Our analysis identified candidate molecular markers for these three nAmb neuron subtypes: *Crhr2, Vipr2*, and *Adcyap1*. The two other clusters appear to have originated from a nearby *Chat*^+^ region, as they express genes found around but not in the nAmb (*Slc17a6* and *Slc6a5*; Figure 1C) (Anderson et al., 2016; Sherman et al., 2015; Tanaka et al., 2003). Based on their molecular differences, we hypothesized that the three nAmb neuron subtypes play distinct physiological roles.

Interestingly, two nAmb subtypes, Crhr2^nAmb^ neurons and Vipr2^nAmb^ neurons, expressed the gene *Calca*, which encodes calcitonin gene-related peptide (CGRP; Figure 1H). CGRP is enriched in esophageal and airway motor neurons in mammals (Lee et al., 1992; McWilliam et al., 1989). To confirm *Calca* expression, we performed FISH and detected *Calca* transcript in approximately 97% of Crhr2^nAmb^ neurons and 49% of Vipr2^nAmb^ (102/105 *Chat*^+^ *Crhr2*^+^ neurons were *Calca*^+^; 115/232 *Chat*^+^ *Vipr2*^+^ neurons were *Calca*^+^; Figures 1I-1K). By comparison, our sNuc-seq analysis detected *Calca* expression in 100% and 70% of the same populations (Figures 1H and 1K). Overall, our sNuc-seq analysis predicts three subtypes of nAmb neurons and suggests that the *Calca*-expressing subtypes, Crhr2^nAmb^ and Vipr2^nAmb^, may be motor neurons for the esophagus and upper airways.

### *Vipr2* and *Crhr2* transcripts mark anatomically distinct nAmb neurons

**Figure 2.**
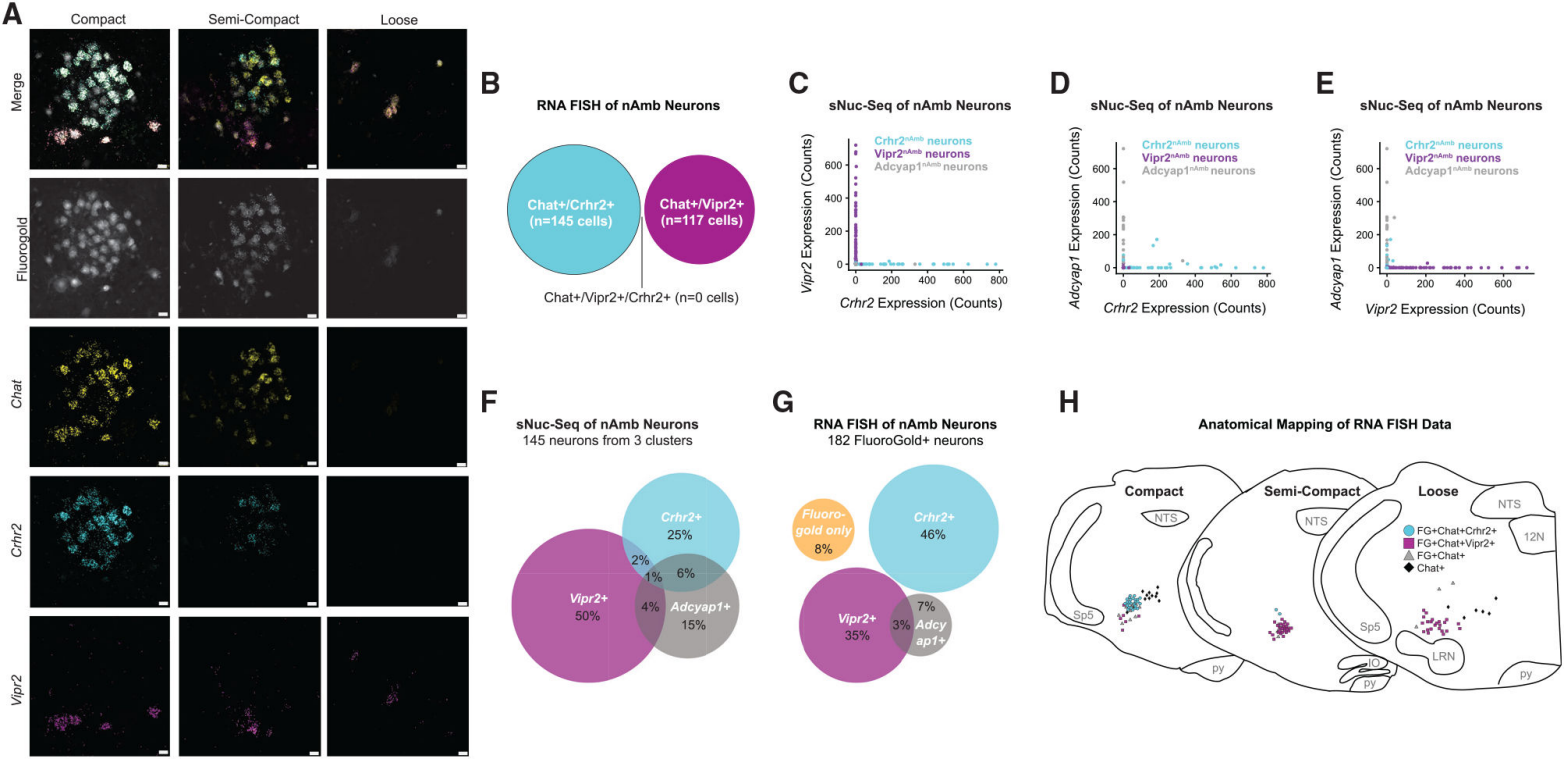
Vipr2 and Crhr2 transcripts mark anatomically distinct subtypes of nAmb neurons (A) Fluorescence *in situ* hybridization (FISH) of *Chat, Crhr2*, and *Vipr2* transcripts in the compact, semi-compact, and loose nAmb, co-localized with systemically administered FluoroGold (n = 3 mice). Scale bar, 20 μm. (B) Venn diagram from FISH data showing lack of cellular colocalization between *Vipr2* transcripts and *Crhr2* transcripts. (C) Co-expression of *Vipr2* and *Crhr2* genes in neurons of the 3 nAmb neuron clusters in the sNuc-seq data. (D) Co-expression of *Adcyap1* and *Crhr2* genes in neurons of the 3 nAmb neuron clusters in the sNuc-seq data. (E) Co-expression of *Adcyap1* and *Vipr2* genes in neurons of the 3 nAmb neuron clusters in the sNuc-seq data. (F) Venn diagram of *Crhr2, Vipr2*, and *Adcyap1* expression among neurons in the 3 nAmb neuron clusters, as detected by sNuc-seq. Expression counts of >1 were considered positive expression. (G) Venn diagram of *Crhr2, Vipr2*, and *Adcyap1* mRNA as detected by RNA FISH (n = 3 mice). nAmb neurons were labeled by systemic injection of the retrograde tracer, FluoroGold. (H) Rostral to caudal distribution of *Chat*^+^/*Vipr2*^+^ and *Chat*^+^/*Crhr2*^+^ neurons throughout the extent of the nAmb (n = 3 mice). DVC, dorsal vagal complex; Sp5, spinal trigeminal nucleus; 12N, hypoglossal nucleus; LRN, lateral reticular nucleus; IO, inferior olivary nucleus; py, pyramidal tract. Compact nAmb: −6.47 mm through −6.75 mm from bregma, Intermediate nAmb: −6.83 mm through −7.10 mm from bregma, loose nAmb: −7.19 mm through −7.46 mm from bregma.

In rodents, nAmb neurons innervating the esophagus, larynx, and pharynx reside in different subregions (Bieger and Hopkins, 1987; Holstege et al., 1983; McGovern and Mazzone, 2010), named based on their relative cell densities as the compact, semi-compact, and loose nAmb (Figure S2E). To map the location of Crhr2^nAmb^ neurons and Vipr2^nAmb^ neurons, we performed FISH for *Vipr2, Crhr2*, and *Chat* mRNA transcripts in the medulla oblongata of wild type mice after labeling all nAmb neurons with systemically administered FG. Essentially all FG-labeled nAmb neurons contained *Chat* mRNA (115/116, or >99% of FG^+^ cells were *Chat*^+^; n = 3 mice; Figure 2A), indicating that all peripherally projecting nAmb neurons are cholinergic and validating our choice of *Chat* to target nAmb neurons for sNuc-seq. Importantly, we observed little to no colocalization of *Vipr2* and *Crhr2* in *Chat*^+^ nAmb neurons (Figure 2B), which agrees with the near lack of *Vipr2* and *Crhr2* co-expression in our sNuc-seq dataset (Figure 2C) and suggests that these genes mark distinct populations of nAmb neurons. Our sNuc-seq analysis detected slightly more overlap in expression of the other markers, between *Adcyap1* and *Crhr2* (Figure 2D) and between *Adcyap1* and *Vipr2* (Figure 2E). Overall, our sNuc-seq analysis of nAmb neurons suggests that *Adcyap1, Vipr2*, and *Crhr2* are expressed by mostly non-overlapping populations of nAmb neurons (Figure 2F).

To validate our sNuc-seq results, we used FISH to visualize *Adcyap1, Vipr2*, and *Crhr2* mRNA in nAmb neurons, as labeled by systemic FG. Our analysis estimates that Crhr2^nAmb^ neurons and Vipr2^nAmb^ neurons constitute 46% and 35% of all nAmb neurons, respectively (n = 3 mice; 182 nAmb neurons total; Figure 2G). The remaining nAmb neurons were either Adcyap1^nAmb^ neurons (7%), *Adcyap1*^+^/*Vipr2*^+^ neurons (3%), or did not express any of the three marker genes (8%). Together, our results indicate that the vast majority of nAmb neurons (89%) express either *Adcyap1, Vipr2*, or *Crhr2* and likely represent distinct subtypes of nAmb neurons.

Mapping the anatomical distribution of Crhr2^nAmb^ neurons and Vipr2^nAmb^ neurons revealed that these subtypes mostly occupy opposite ends of the nAmb (n = 3 mice). The Crhr2^nAmb^ neurons were enriched in the compact and semi-compact nAmb (Figures 2A and 2H), where most esophageal and pharyngeal motor neurons reside (Bieger and Hopkins, 1987; McGovern and Mazzone, 2010). However, Vipr2^nAmb^ neurons were found predominantly in the semi-compact and loose nAmb (Figures 2A and 2H), suggesting that they may be pharyngeal and laryngeal motor neurons (Bieger and Hopkins, 1987; McGovern and Mazzone, 2010).

### Crhr2^nAmb^ neurons and Vipr2^nAmb^ neurons separately innervate the esophagus and upper airways

**Figure 3.**
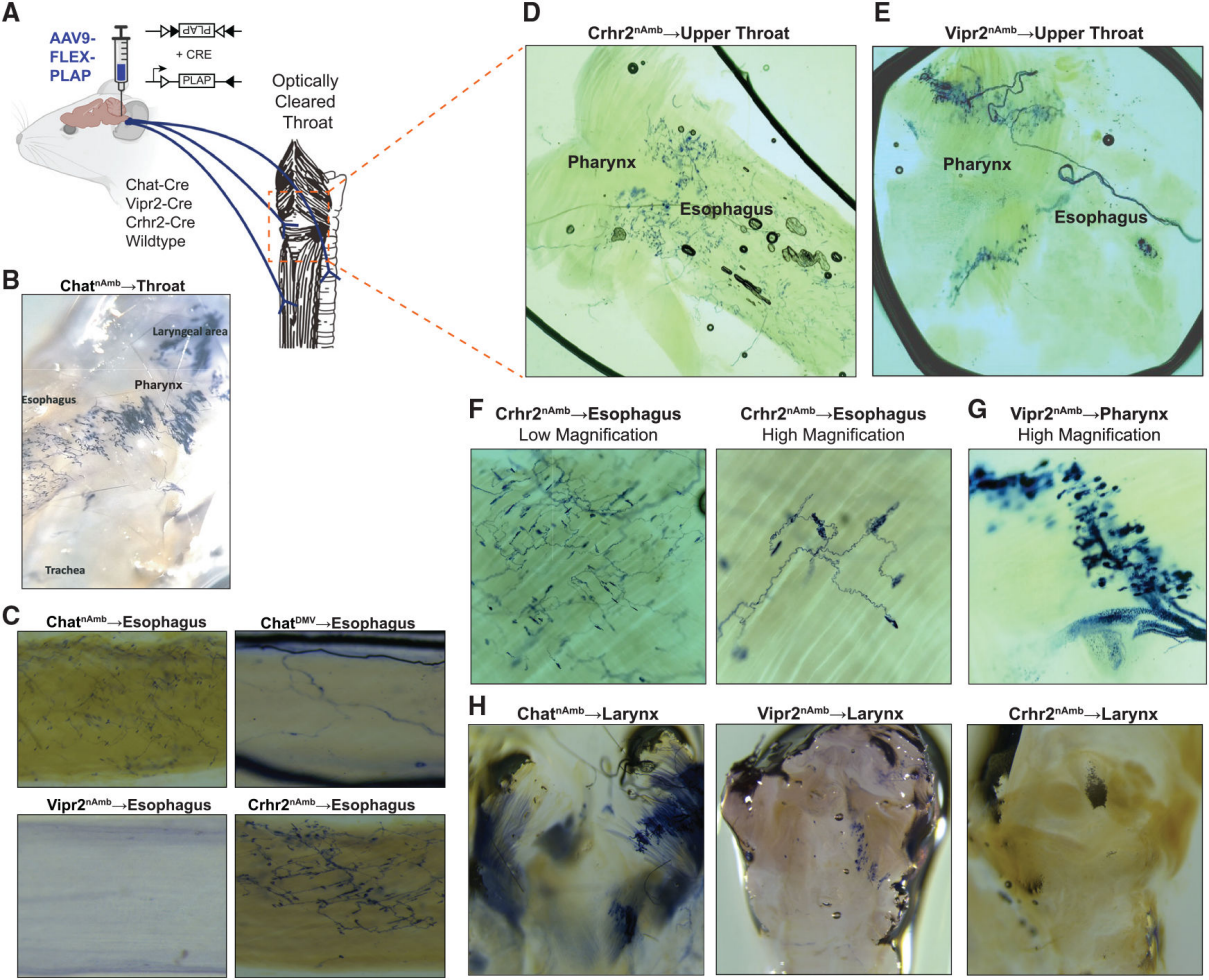
Crhr2^nAmb^ neurons and Vipr2^nAmb^ neurons separately innervate esophagus and pharynx (A) Schematic showing injection of AAV9-FLEX-PLAP into the nAmb of Chat-Cre (n = 6 mice), Vipr2-Cre (n = 5 mice), Crhr2-Cre (n = 5 mice), and wild-type (WT) mice (n = 3 mice). Portions of this figure were created with BioRender.com. (B) PLAP-stained axons in the upper throat after injection in the nAmb of Chat-Cre mice. (C) PLAP-stained axons in the cervical esophagus after injections in the nAmb of Chat-Cre, Vipr2-Cre, Crhr2-Cre, and the DMV of Chat-Cre mice. (D and E) PLAP-stained axons in the lower pharynx and upper esophagus after injection into the nAmb of Crhr2-Cre mice (D) and Vipr2-Cre (E) mice. (F) Low and high magnification images of PLAP-stained axon terminals in the mid-esophagus after injection into the nAmb of Crhr2-Cre mice. (G) High-magnification image of PLAP-stained axons in the pharyngeal muscles after injection into the nAmb of Vipr2-Cre mice. (H) PLAP-stained axon terminals in the larynx after injection into the nAmb of Chat-Cre, Vipr2-Cre, and Crhr2-Cre mice.

Given the distinct molecular and spatial profile of Crhr2^nAmb^ and Vipr2^nAmb^ neurons, we investigated whether these nAmb subtypes also differ in which tissues they innervate. To map the axon projections of the nAmb, we injected an AAV that Cre dependently expresses placental alkaline phosphatase (PLAP; AAV-FLEX-PLAP]) into the ventrolateral medulla of Cre driver mouse lines (n = 6 mice; Figure 3A; Prescott et al., 2020). We first established that this approach labels nAmb neurons by injecting AAV-FLEX-PLAP in Chat-Cre mice. Following recovery, PLAP immunofluorescence was detected in 63% of ChAT immunoreactive neurons in the nAmb area (98 ChAT^+^/PLAP^+^ out of 155 ChAT^+^ neurons, n = 2 mice; Figure S2A) distributed along the rostro-caudal extent of the nucleus. PLAP^+^ cholinergic neurons were also observed in the caudal aspect of the facial motor nucleus and cuneiform nucleus, but not in the dorsal motor nucleus of the vagus (DMV), another major source of vagal motor neurons, or the hypoglossal nucleus, which innervates the tongue. Injecting AAV-FLEX-PLAP in Cre^−^ mice resulted in no detectable PLAP expression in the brainstem (Figure S2B). Together, this evidence indicates that our injection of AAV-FLEX-PLAP into the ventrolateral medulla efficiently labels most nAmb neurons while avoiding labeling of the DMV and hypoglossal nucleus, and that PLAP expression is limited to Cre-expressing cells.

To visualize the axons of PLAP^+^ neurons, we performed a reaction to stain the PLAP^+^ axons dark blue and then optically cleared the esophagus, airways, heart, lungs, and tongue (Prescott et al., 2020). Targeting PLAP to all nAmb neurons (Chat^nAmb^) labeled axons throughout the larynx, pharynx, and esophagus (n = 6 mice; Figure 3B), as well as sparse fibers on the trachea (Figures 3B and S2C). We observed no PLAP-stained axons from Chat^nAmb^ neurons to the tongue, consistent with previous retrograde tracing studies showing that the nAmb does not innervate the tongue in mouse (Stanek et al., 2014). Unexpectedly, however, we observed few to no PLAP-labeled axons from Chat^nAmb^ neurons to the lungs or heart (Figure S2C), major targets of nAmb parasympathetic neurons, indicating that our methods were not suitable for visualizing nAmb innervation of these organs. Together, our anterograde tracing studies confirm that nAmb neurons innervate the larynx, pharynx, esophagus, and trachea.

The DMV is another major source of vagal motor neurons, although it is thought primarily to innervate subdiaphragmatic digestive organs. To characterize DMV projections to the cervical esophagus in mice, we repeated our PLAP studies but instead targeted Chat-Cre^+^ DMV neurons (Chat^DMV^; n = 3 mice). Our results showed that PLAP-stained Chat^DMV^ fibers branch extensively in the stomach wall (Figure S2D), but not in the cervical esophagus (Figure 3C). Stained nerves and fiber bundles from Chat^DMV^ neurons were evident along the esophagus, but lacked the branching pattern and motor endplate morphology of the Chat^nAmb^ fibers (Figure 3C). These results confirm that the nAmb is the predominant source of motor innervation of the cervical esophagus in mice (Sang and Young, 1998).

To map the projections of Crhr2^nAmb^ neurons and Vipr2^nAmb^ neurons, we obtained Crhr2-Cre and Vipr2-Cre mice. We validated Cre expression in these mouse lines by co-localizing mRNA of the driver gene (i.e., *Crhr2* or *Vipr2*) and *Cre* using FISH. In Crhr2-Cre mice (n = 3 mice), 79/82 *Crhr2*^+^ neurons were also *Cre*^+^ (representative image in Figure S3C). In Vipr2-Cre mice (n = 4 mice), 57/57 *Vipr2*^+^ neurons were *Cre*^+^ (representative image in Figure S3D). Importantly, in the hindbrain of both Cre mouse lines, Cre mRNA was limited to cells in which the driver gene mRNA was also detected, confirming the specificity of these Cre lines (Figures S3C and S3D). We then injected AAV-FLEX-PLAP into the ventrolateral medulla of *Crhr2*-Cre mice and *Vipr2*-Cre mice. This resulted in patterns of PLAP immunofluorescence that were consistent with the anatomical distribution of Crhr2^nAmb^ neurons and Vipr2^nAmb^ neurons (Figures 2H, S3A, and S3B). We observed no PLAP immunofluorescence in the DMV or the hypoglossal, suggesting that our injections did not reach these regions. While we did observe PLAP labeling in some neurons around the nAmb, none of these neurons were FG^+^, indicating that they do not project peripherally and thus should not confound our anterograde tracing studies. These results suggest that the only peripherally projecting neurons expressing PLAP were nAmb neurons.

We then visualized innervation by Crhr2^nAmb^ and Vipr2^nAmb^ neurons in the esophagus and upper airways. Stained fibers from Crhr2^nAmb^ neurons formed a dense plexus of terminations in the cervical and upper esophagus, but few to none were visible in the upper pharynx, larynx, or trachea (Figures 3C, 3D, 3H, and S3E). The esophageal Crhr2^nAmb^ fibers formed sparse motor endplates along perpendicular axes (Figure 3F), suggesting that they innervate both circular and longitudinal muscles (Powley et al., 2013). In striking contrast, Vipr2^nAmb^ fibers did not visibly innervate the esophagus, tongue, trachea, heart, or lungs, but did branch extensively in the pharynx (Figures 3C, 3E, and S3F), where they formed grape-like clusters of terminals in bands along an axis perpendicular to the muscle fibers (Figure 3G), consistent with the structure of pharyngeal nerve fibers in rats (Kobler et al., 1994). When we imaged the larynx, we observed stained fibers from Chat^nAmb^ neurons and Vipr2^nAmb^ neurons but not from Crhr2^nAmb^ neurons (Figure 3H). Collectively, these results indicate that the Crhr2^nAmb^ neurons and Vipr2^nAmb^ neurons differentially innervate the esophagus and upper pharynx and larynx, respectively.

### Activating Crhr2^nAmb^ neurons affects esophageal motor activity but not heart rate

**Figure 4.**
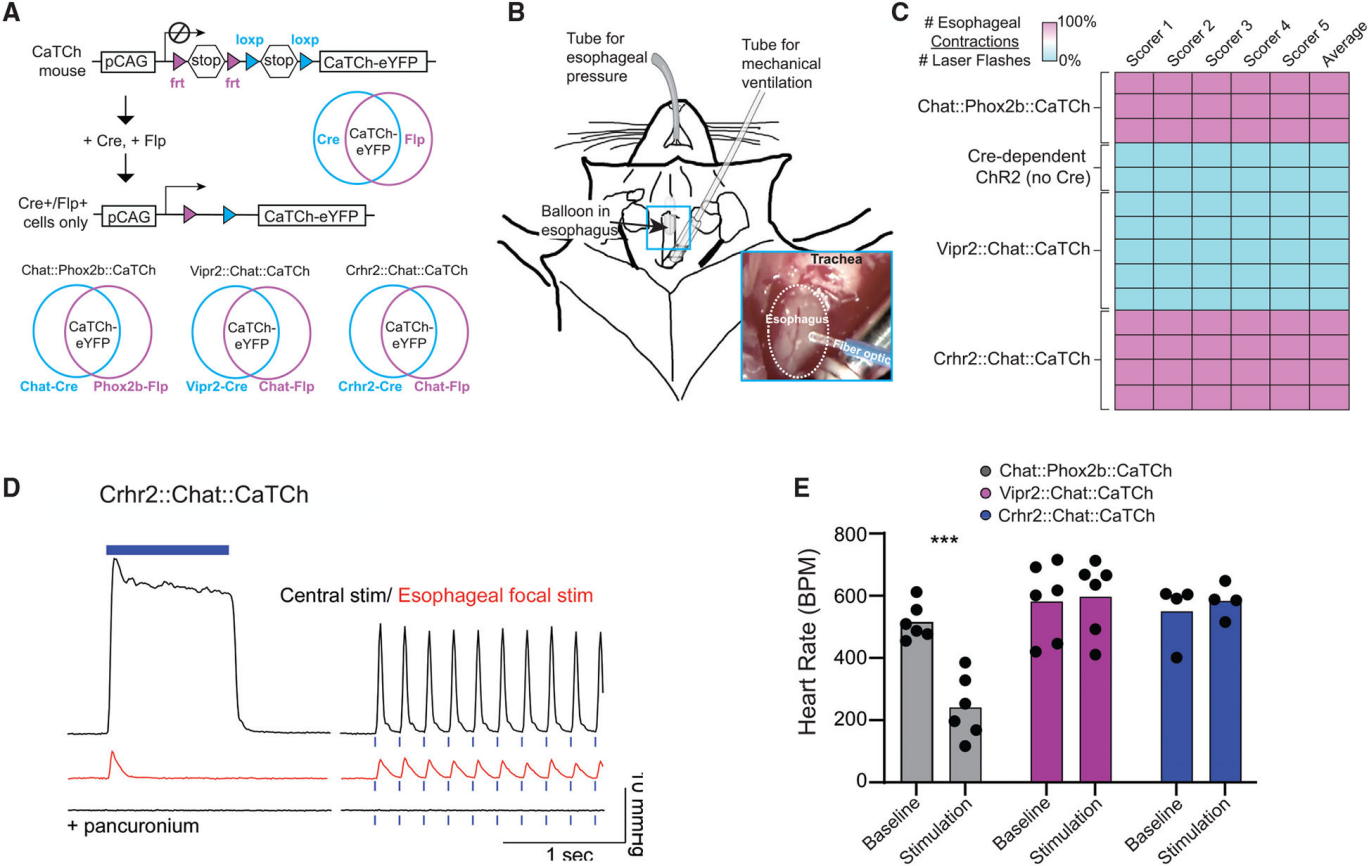
Crhr2^nAmb^ neurons selectively control esophageal muscles (A) Intersectional optogenetics approach for activating all nAmb neurons, Vipr2^nAmb^ neurons, and Crhr2^nAmb^ neurons. CaTCh-EYFP expression is dependent on the presence of both Cre and Flp recombinases. (B) Schematic of experimental setup for measuring esophageal function. The mouse is anesthetized and mechanically ventilated to expose the esophagus. A balloon is inserted in the esophagus to measure esophageal pressure and a fiber optic is placed above the esophagus for focal stimulation. (C) Blinded scoring of video data from optogenetic stimulation of esophagus in different genotype groups. Values represent the number of visible muscle contractions divided by the number of laser flashes observed during the video. (n = 3–4 mice per genotype, 2 mice for negative control). (D) Representative pressure recordings from balloon pressure transducer in esophagus during photostimulation of Crhr2-Cre:Chat-Flp:CaTCh mouse via optical fiber either implanted in nAmb (“central”) or placed over the esophagus. Esophageal response before and after administration of pancuronium, a competitive inhibitor of nicotinic acetylcholine receptors at the neuromuscular junction. (E) Heart rate before and after photostimulation of Chat-Cre:Phox2b-Flp:CaTCh (n = 6 mice), Vipr2-Cre:Chat-Flp:CaTCh (n = 6 mice), and Crhr2-Cre:Chat-Flp:CaTCh (n = 4 mice) mice via nAmb-implanted optical fiber (***p < 0.001 for baseline versus stimulation by Sidak’s multiple comparison test; F = [2,26] = 8.538; p = 0.0014).

Since Crhr2^nAmb^ neurons selectively innervate the esophagus, we hypothesized that they control esophageal motor activity. To investigate this, we used intersectional optogenetics to activate nAmb subtypes while imaging motor responses in the esophagus. First, we intersectionally defined Crhr2^nAmb^ neurons and Vipr2^nAmb^ neurons by crossing Crhr2-Cre mice and Vipr2-Cre mice to a knockin Chat-Flp mouse line, which we validated by co-localizing *Chat* mRNA with *Flp* mRNA by FISH (n = 3; 121/121 *Chat*^+^ cells in the hindbrain were *Flp*^+^; Figure S4A). Second, we bred the Cre:Flp mice to a transgenic intersectional mouse line that expresses Cre- and Flp-dependent CatCh, a calcium-permeable channelrhodopsin2 (Daigle et al., 2018). We used the offspring to optogenetically activate different nAmb neuron populations as follows: all nAmb neurons using Chat-Cre:Phox2b-Flp:CaTCh mice (Chat:Phox2b:CaTCh), Vipr2^nAmb^ neurons using Vipr2-Cre:Chat-Flp:CaTCh mice (Vipr2:Chat:CaTCh), and Crhr2^nAmb^ neurons using Crhr2-Cre:Chat-Flp: CaTCh mice (Crhr2:Chat:CaTCh; Figure 4A and S4B-S4E). As a negative control, we also included a group of mice that Cre dependently expressed ChR2 (Ai32 mice), but which were not crossed to a Cre-expressing mouse line.

We performed focal photostimulation of the ventral surface of the esophagus in anesthetized, mechanically ventilated mice (Figure 4B). This approach minimizes the chances of activating non-nAmb neurons because cholinergic nAmb neurons are the principal source of motor innervation of the esophagus. Focal stimulation of the esophagus in Crhr2:Chat:CaTCh mice (n = 4) produced time-locked movements localized to the site of stimulation that could be reliably identified in video recordings by blinded scorers. A similar result was obtained in experiments in which all nAmb were targeted (Chat:Phox2b:CaTCh; n = 3). Conversely, focal stimulation of the esophagus had no visible effect in Vipr2:Chat:CaTCh mice (n = 4) or in negative controls (Figure 4C; n = 2). To assess whether the movements evoked by photostimulation of the esophagus in Crhr2:Chat:CaTCh mice reflected constriction, we measured esophageal pressure during photostimulation with a balloon catheter. We photostimulated both focally in the esophagus and centrally through a fiber optic implanted over the nAmb (Figure S4F) in the same mice. With both approaches, photostimulation resulted in abrupt increases in esophageal pressure that were frequency dependent and time locked to the pattern of photostimulation, with the effects of central stimulation being more robust (Figures 4D and S4G; n = 6). When the balloon catheter was retracted into the mouth, focal stimulation of the esophagus no longer produced detectable changes in pressure supporting the conclusion that focal stimulation results in contractions that are generated locally within the esophagus. Pressure changes were recorded in the mouth during central stimulation in Crhr2:Chat:CaTCh mice (Figure S4G), which may be related to movements in the mouth secondary to strong contractions of the esophagus and/or the central activation of the axons of facial and hypoglossal motor neurons that express CaTCh^+^ in this mouse line. Pancuronium, a competitive inhibitor of nicotinic acetylcholine receptors, blocked the increases in esophageal pressure that occurred with both central and focal esophageal stimulation of Crhr2:Chat:CaTCh neurons (Figure 4D; n = 2). These results indicate that *Crhr2* labels cholinergic esophageal motor neurons that control the cervical esophagus through nicotinic receptor signaling.

Since we were unable to visualize nAmb projections to the heart with the PLAP method, we assessed the effects of stimulating nAmb neurons on heart rate in freely behaving mice instrumented to record electrocardiogram (EKG). We targeted Chat^nAmb^ neurons (n = 6 mice), Vipr2^nAmb^ neurons (n = 6 mice), or Crhr2^nAmb^ neurons (n = 4 mice) by implanting a fiber optic above the right nAmb in the corresponding Cre:Flp:CaTCh lines. Stimulating the nAmb in Crhr2:Chat:CaTCh or Vipr2:Chat:CaTCh mice resulted in small and variable changes in heart rate that were not different from negative controls, whereas stimulating all Chat^nAmb^ neurons using Chat:Phox2b:CaTCh mice immediately and significantly decreased the heart rate by nearly half compared to baseline (Figure 4E). The decrease in heart rate observed when activating all of the nAmb neurons (Chat^nAmb^) is likely due to a nAmb neuron subtype that expresses *Chat* but not *Vipr2* or *Crhr2* (e.g., Adycap1^nAmb^ neurons; Figures 1B-1D). These results suggest that Crhr2^nAmb^ and Vipr2^nAmb^ neurons are not cardiovagal neurons. Of note, central stimulation produced time-locked whisking and movements localized to the head and neck in all of the Cre:Flp:CaTCh lines, but not in negative controls. The presence of oro-facial motor responses in these experiments suggests that central stimulation activates cranial motor neurons outside of the nAmb, specifically in the hypoglossal and facial motor pools. Collectively, our data suggest that the nAmb comprises genetically defined neuron subtypes that play distinct physiological roles. Of these neuron subtypes, our results indicate that Crhr2^nAmb^ neurons selectively innervate the esophagus and are capable of contracting esophageal muscles.

## DISCUSSION

The nAmb is the primary source of motor input to the larynx, pharynx, and cervical esophagus and of parasympathetic input to the heart, lungs, and airways. Combining single-cell transcriptomics, anterograde tracing, and intersectional optogenetics, our results identify three subtypes of nAmb neurons and suggest that one subtype, Crhr2^nAmb^ neurons, are esophageal motor neurons. Crhr2^nAmb^ neurons innervate the esophagus, and when activated, reliably contract the esophagus but do not affect the heart rate. While previous studies showed that esophageal motor neurons occupy a distinct subregion of the nAmb, this study defines the molecular organization of these motor neurons and links it with their anatomy and function.

The molecular profiles of esophageal motor neurons may reveal insight into their physiology and function. For instance, morphine increases the rate of esophageal peristalsis in humans (Penagini et al., 1996), and our results show that the gene encoding the morphine receptor *Oprm1* is highly enriched in Crhr2^nAmb^ neurons (Figure 1G). The gene expression profile for Crhr2^nAmb^ neurons provided by our study, including many receptors, neuropeptides, and other signaling proteins, can be mined for pharmacological targets to treat diseases such as esophageal motility disorder.

Together with a recent study by Tao et al. (2021), our results support a “labeled line” organization of vagal motor neurons. Tao et al. identify seven molecular subtypes of vagal motor neurons in the DMV, the major source of parasympathetic input to the gut. Two DMV subtypes specifically innervate the glandular stomach but target neurochemically distinct enteric neurons (Tao et al., 2021). These findings raise the possibility that the functional units of the DMV (Huang et al., 1993) are genetically defined. Our study suggests a similar organization of the nAmb, another vagal motor nucleus. Interestingly, genetic coding of cellular function is also true of vagal sensory neurons, which show a clear correspondence between molecular subtype and physiological role (Bai et al., 2019; Borgmann et al., 2021; Chang et al., 2015; Kupari et al., 2019; Prescott et al., 2020; Williams et al., 2016). Furthermore, sympathetic preganglionic neurons in the spinal cord comprise transcriptionally diverse subtypes as well (Blum et al., 2021), raising the possibility that functionality may be genetically encoded throughout the autonomic nervous system.

Our study raises several questions for further investigation. For instance, what is the physiological role of Crhr2^nAmb^ neurons? Our results indicate that they are capable of contracting esophageal muscle through nicotinic receptor signaling. Does this represent physiological control and is it necessary for esophageal function? Also, are all Crhr2^nAmb^ neurons capable of contracting the esophagus, or just a subset? Our sNuc-seq analysis relied on 145 nAmb neurons, or only ~15% of the neurons present in 1 hemisphere of the adult mouse nAmb (Sturrock, 1990). While our FISH analysis shows that 92% of peripherally projecting nAmb neurons express *Crhr2, Vipr2*, and/or *Adcyap1*, our sNuc-seq analysis may underestimate the true diversity of neuron subtypes in the nAmb. For instance, although our anterograde tracing data show that Crhr2^nAmb^ neurons innervate the esophagus, Crhr2^nAmb^ neurons may comprise subtypes, one or more of which could be esophageal motor neurons.

### Conclusions

Overall, our study provides three major advances to understanding the neural control of esophageal function: (1) identifies the primary motor neurons for the esophagus molecularly, anatomically, and functionally; (2) reveals a genetic logic for the functional organization of the nAmb; and (3) comprehensively characterizes the gene expression profile of esophageal motor neurons, which can be mined for potential drug targets to treat swallowing disorders.

### Limitations of the study

Our sNuc-seq analysis includes only male mice. Both males and females were used in our other studies, including the FISH validation of Crhr2^nAmb^ and Vipr2^nAmb^ subtypes, suggesting that the same subtypes present in male nAmb are also present in female nAmb. However, our results cannot exclude the possibility that additional neuron subtypes exist in the female nAmb.

Our study is mostly qualitative. For instance, our functional studies assessed whether activating cholinergic Crhr2^+^ axons in the esophagus caused contractions but not whether the magnitude or timing of these contractions were within a physiological range. More quantitative studies and those involving loss of function are needed to fully understand the role of Crhr2^nAmb^ neurons.

Our functional data rely on targeting neurons in the nAmb using the intersection of genes that are not restricted to the nAmb. *Vipr2* and, to a lesser extent, *Crhr2*, are expressed in cholinergic neurons supplying the facial and hypoglossal nerves. Consistent with this, movements of the tongue and whiskers were clearly visible during central optogenetic stimulation targeting the cell bodies in the nAmb regardless of the particular combination of neurons targeted. During central stimulation of Crhr2^nAmb^ neurons, the stimulation of neurons that contract the tongue in particular confounds the interpretation of esophageal pressure changes with this approach. However, the effects of focal stimulation reflect changes that are generated only by axons in the cervical esophagus. Based on previous studies, these cholinergic axons principally arise from esophageal motor neurons in the nAmb (Sang and Young, 1998). Hence, focal stimulation of the cervical esophagus provides a reasonably selective approach for identifying neurons with a motor function in this region. A more definitive dissection of esophageal motor circuitry would require nAmb-specific opsin expression.

Our results do not rule out the possibility that photoactivating the esophagus in Crhr2:Chat:CaTCh and Chat:Phox2b:CaTCh mice contracted the esophagus through indirect effects on other tissues. However, it is unlikely the esophageal contractions were secondary to effects on tongue muscles since the tongue is not known to be innervated by the nAmb (Jean, 2001; Stanek et al., 2014) and since our anterograde tracing with PLAP failed to detect tongue innervation by Chat nAmb neurons (Figure S4). Also, it is unlikely that the esophageal contractions we observed were due to effects on the lower esophageal sphincter (LES), since studies in ferrets have demonstrated that changes in LES pressure do not trigger esophageal contractions (Abrahams et al., 2002). Direct activation of esophageal neuromuscular circuits is therefore the most parsimonious explanation for the esophageal contractions we observed when photostimulating the esophagus in Crhr2:Chat:CaTCh and Chat:Phox2b:CaTCh mice.

## STAR★METHODS

### RESOURCE AVAILABILITY

#### Lead contact

Further information and requests for resources and reagents should be directed to and will be fulfilled by the lead contact, John Campbell (jnc4e@virginia.edu).

#### Materials availability

- The *Chat*-p2A-Flp mouse generated in this study will be made available on request.
- Requests for *Chat*-p2A-Flp mouse should be made to Bradford B. Lowell, M.D., Ph.D., Department of Endocrinology, Diabetes, and Metabolism, Beth Israel Deaconess Medical Center and Harvard Medical School, blowell@bidmc.harvard.edu.

#### Data and code availability

- The accession number for the raw and processed sNuc-seq data and metadata reported in this paper is GEO accession number GSE202760.
- A user-friendly interface for visualizing and exploring the sNuc-seq data is available at the Broad Institute Single Cell Portal at https://singlecell.broadinstitute.org/single_cell/study/SCP1677.
- The code used for processing, clustering, and visualizing the clustered sNuc-seq data is publicly available through Zenodo at https://doi.org/10.5281/zenodo.6564316.
- Any additional information required to reanalyze the data reported in this paper is available from the lead contact upon request.

### EXPERIMENTAL MODEL AND SUBJECT DETAILS

All animal care and experimental procedures were approved in advance by the University of Virginia Institutional Animal Care and Use Committee. The sNuc-Seq experiments used adult male Chat-Cre mice (Jackson Laboratories, JAX, stock # 28861; 28 weeks old). The fluorescent *in situ* hybridization experiments used C57BL/6J mice from the Jackson Laboratory (JAX, 000664). The following mouse lines were used for optogenetic studies: Chat-Cre (Rossi et al., 2011; JAX, 028861); Crfr2α-eGFPCre bacterial artificial chromosome (BAC) transgenic mice (“Crhr2-Cre mice”; Anthony et al., 2014); Vipr2-Cre (Daigle et al., 2018; JAX, 31332); Phox2b-Flp (Hirsch et al., 2013; JAX, 22407); CaTCh (Daigle et al., 2018; JAX, 025109); Ai32 (RCL-ChR2(H134R)/EYFP) mice (Madisen et al., 2012; JAX, 024109); and a Chat-p2a-Flp mouse line (see Method Details below). The anterograde tracing experiments used Chat-Cre, Crhr2-Cre, Vipr2-Cre, and C57BL/6J mice. Unless otherwise specified, all experiments used adult mice with approximately equal numbers of male and female mice. Mice were housed at 22-24°C with a 12-h light:12-h dark cycle and *ad libitum* access to standard mouse chow and water.

### METHOD DETAILS

#### Chat-p2a-Flp mouse

Chat-p2a-Flp mice were generated using Easi-CRISPR method (Miura et al., 2018). Briefly, a single-stranded DNA donor containing the p2a-Flp cassette, flanked by 100-base homology arms both up- and down-stream of the *Chat* stop codon was designed. A single guide RNA (sequence: 5′-CCCACTAGCCAATGTCCTAC-3′) was designed to induce the cut in the genome. For pronuclear injection, ssDNA donor, sgRNA and *Cas9* protein were co-injected into mouse fertilized eggs of FVB strain. Live-born pups were screened by PCR reactions, and the positives were then sequenced to confirm the correct insertion.

#### Single-nuclei RNA-Seq

The ventrolateral medulla of five male Chat-Cre mice were injected with a Cre-dependent reporter virus, AAV-DIO-H2b-mCherry. Four weeks later, at the age of 27 weeks, mice were rapidly decapitated for brain extraction to avoid stress related changes in nuclear mRNA. Following immediate brain extraction, 1 mm thick coronal sections of hindbrain through the nAmb’sfull rostral-caudal extent (Bregma −6.5 mm to −8.0 mm) were cut and immersed in ice-cold RNA-later (Qiagen catalog # 76106). After at least 30 min in ice-cold RNA-later, the nAmb was visualized under a fluorescence stereomicroscope (Zeiss Discovery V8) and dissected, then stored in RNA-later overnight at 4°C. On the next day, nAmb tissue was homogenized and purified by density-gradient centrifugation into a single-nuclei suspension as previously described (Habib et al., 2016; Todd et al., 2020). The single-nuclei suspension was sorted by FACS to isolate one H2b-mCherry+ nucleus per well of three 96-well plates, which were then centrifuged at 2,500rcf, frozen on dry ice, and stored at −80°C until use. After purifying the RNA by RNA-clean SPRI reagents (1.5x ratio of SPRI to sample), cDNA libraries were generated from polyadenylated mRNA of each sample using Smart-Seq2 (Picelli et al., 2014), modified as described previously (Tao et al., 2021). Illumina sequencing libraries were made from single-nuclei cDNA samples as described previously (Tao et al., 2021) and then sequenced by Illumina Next-Seq 500. Reads were demultiplexed by bcl2fastq2 v2.20.0 (Illumina) and aligned to the mouse genome by STAR v2.6.1 (Dobin et al., 2013). Duplicates were removed with Picard Tools v2.18.21. Aligned reads were processed into a digital gene expression (DGE) file with Drop-Seq Tools v2.3.0 and tagged using “GENCODE_M16_PRI” annotation. Since the sNuc-seq protocol does not produce true UMIs, we considered any unique read (i.e., non-duplicated) as a unique “UMI-tagged” read for the Drop-Seq pipeline. An R software package for single-cell genomics analysis, Seurat v4.0 (Hao et al., 2021), was used to filter, scale, and normalize the data, then perform dimensionality reduction with principal component (PC) analysis on the top 2,000 most variable genes, cell clustering on the first 6 PCs, and differential expression analysis using Wilcoxon Rank Sum test and default settings, as previously described (Tao et al., 2021; Todd et al., 2020). Low quality samples (<2000 genes detected per nucleus) were excluded from this analysis. Transcription factor and receptor gene lists were derived from the Panther v16.0 protein and gene classification site, http://www.pantherdb.org/ (Mi et al., 2021).

#### Fluorescence *in situ* hybridization (FISH)

FISH experiments were performed on brain tissue from mice that received one intraperitoneal injection of 2% Fluorogold (Fluorochrome) a minimum of 5 days prior to euthanasia. Mice were terminally anesthetized with ketamine (20 mg/kg) and xylazine (2 mg/kg) diluted in PBS, followed by transcardial perfusion with 0.9% saline plus heparin and 4% paraformaldehyde (Thomas Scientific). Brains were extracted and post-fixed for 24 h at 4°C. Following fixation, brains were sectioned coronally at 30–35 um thickness on a vibratome (Leica). The day before FISH, the sections were rinsed in PBS and then mounted on slides (Fisher) and left to dry overnight. An ImmEdge Hydrophobic Barrier Pen was used to draw a barrier around the sections. The sections were then incubated in Protease IV in a HybEZ II Oven for 30 min at 40°C, followed by incubation with target probes (*Chat, Vipr2, Crhr2, Calca, Adcyap1*, and *Phox2b*) for 2 h at 40°C. Slides were then treated with AMP 1–3, HRP-C1, HRP-C2, HRP-C3, and HRP Blocker for 15–30 min at 40°C, as previously described (Wang et al., 2012). FITC, Cy3, and Cy5 (Perkin Elmer) were used for probe visualization. Fluorogold was either imaged in its native state, or visualized using immunofluorescence with a rabbit anti-Fluorogold primary antibody (Fluorochrome, 1:5000) overnight and a donkey anti-rabbit 647 secondary antibody (Thermo Scientific Cat# A31573, Lot# 2083195, 1:1000) for 2–4 h. Images were taken using a confocal microscope (Zeiss). Cell distributions were mapped in Neurolucida software (version 11, MBF Bioscience) using an AxioImager M2 (Carl Zeiss). Composite maps of nAmb neurons reflect cells in 3 hemi-sections of the brainstem spanning 270 um in the rostro-caudal axis overlaid on a single wireframe representing the three levels of the nAmb. The three levels of the NAmb correspond to the following bregma levels according to Paxinos and Franklin (2013) (Compact nAmb: −6.47 mm through −6.75 mm from bregma, Intermediate NAmb: −6.83 mm through −7.10 mm from bregma, loose nAmb: −7.19 mm through −7.46 mm from bregma).

#### Anterograde tracing viral injections

Mice were anesthetized with ketamine (20 mg/kg) and xylazine (2 mg/kg) diluted in PBS and positioned into a stereotaxic apparatus (Kopf). A pulled glass micropipette was used for stereotaxic injections of an adeno-associated virus vector, AAV9-CAG-FLEX-PLAP (Prescott et al., 2020), a gift of Dr. Stephen Liberles (Harvard Medical School, Howard Hughes Medical Institute), using the following stereotaxic coordinates for the nAmb: anterior/posterior −2.1, −2.4, −2.7 mm, lateral/medial +/− 1.3 mm, and dorsal/ventral −5.8 mm, from lambda; and anterior/posterior −0.1 mm, lateral/medial +/− 1.3 mm, and dorsal/ventral −0.1, −1.3 mm, from the calamus scriptorius. Following local anesthetization with bupivacaine, virus was injected (200 nL/injection, 2–3 injections/side) using a Nanoject III system. This injection strategy was designed to fully cover the nAmb, not to restrict viral infection to only nAmb neurons. The pipette was removed 3–5 min after injections, followed by wound closure using sutures or surgical wound glue (Vetbond). Meloxicam SR (5 mg/kg; sustained release, SR) was injected subcutaneously for post-operative analgesia.

#### Placental alkaline phosphatase staining

Following 3-4wk after AAV9-CAG-FLEX-PLAP injection, mice were terminally anesthetized with ketamine (20 mg/kg) and xylazine (2 mg/kg) diluted in PBS, followed by transcardial perfusion with 0.9% saline plus heparin then 4% paraformaldehyde (Thomas Scientific). The brains, esophagus, trachea, larynx, and lungs were collected and post-fixed for 24 h at 4°C. Following fixation, brains were sectioned coronally at 30 or 35 um thickness on a vibratome (Leica). A single series of sections per animal was used in histological studies to confirm injection site and PLAP expression. All subjects determined to be surgical “misses” based on little or absent reporter expression were excluded from analyses. The esophagus, lungs, upper airways, heart, and tongue were washed three times for 1 h at room temperature in PBS, followed by incubation in alkaline phosphatase (AP) buffer (0.1 M Tris HCl pH 9.5, 0.1 M NaCl, 50 mM MgCl2, 0.1% Tween 20, 5 mM tetramisole-HCl) for two hours at 70°C. Afterward, the samples were equilibrated to room temperature and then washed twice in AP buffer. AP activity was visualized with NCT/BCIP solution (ThermoFisher Scientific 34042) and stained samples were rinsed in AP buffer for 15 min, post-fixed in 4% PFA for 1 h, and washed in PBS. Samples were then dehydrated through a series of ethanol washes (15%–100%) and cleared using a 1:2 mixture of benzyl alcohol (Sigma-Aldrich 402834-500 ML) and benzyl benzoate (Sigma-Aldrich B6630-1L). Whole mount images were taken using a brightfield stereomicroscope (Leica M205 FCA Stereomicroscope with color camera) and fluorescence microscope (Echo Revolve).

#### Optogenetic physiology

Cre-expressing mouse lines were crossed to Chat-Flp or Phox2b-Flp mouse lines to generate Cre::Flp lines, which were then crossed to transgenic mice that express eYFP tagged calcium-permeable channelrhodopsin 2 (CaTCh) only after recombination by both Cre and Flp recombinases. Mice from these Cre::Flp::CaTCh lines were implanted with an optical fiber over the nAmb.

Optical fibers for central stimulation and headsets to record electrocardiogram (ECG) were implanted under anesthetized with ketamine (150 mg/kg) and dexmedetomidine (1 mg/kg). Depth of anesthesia was assessed by absence of the corneal and hind-paw withdrawal reflex. Body temperature was maintained at 37.2 ± 0.5°C with a servo-controlled temperature pad (TC-1000; CWE). Following confirmation of anesthesia, mice were prepped for surgery, positioned in a stereotaxic headframe, and the local anesthetic, Bupivacaine (50 ul of 5 mg/mL), was injected at surgical sites. The tissue overlying the dorsal surface of the skull was retracted and the surface of the skull prepped for headset and optical fiber implants.

EKG headset were constructed from 4-pin miniature connectors soldered to 2 lengths of Teflon-coated multi-strand stainless steel wire (AM-systems) for positive and negative leads and a stainless steel screw implanted in the skull for the ground. The screw was implanted above the frontal cortex and the leads were tunneled subcutaneously to opposing locations near the base of the ribcage. An optical fiber cannula constructed (200um 0.39NA fiber, Thorlabs) was implanted to stimulate cell bodies in the nAmb using the following coordinates from Lambda: Anterior/posterior: −2.1 mm, medial/lateral: +1.3 mm, dorsal/ventral: −5.0 mm for Crhr2-Cre::Chat-Flp::CaTCh mice. Fibers were only implanted on the right side of the brain. The same coordinates were used for Vipr2-Cre::Chat-Flp::CaTCh mice, but at a 10° angle to access these neurons, which are found in the caudal nAmb. The fiber and ECG headset was secured to the skull with dental cement and wounds were closed with sutures and surgical glue. Mice were given ketoprofen (5 mg/kg) for 3 days after surgery and allowed to recover for a minimum of 5 days before optogenetic physiology experiments.

To assess the cardiac effects of stimulating nAmb neurons without anesthesia, mice were scruffed to connect the ECG head set and implanted fiber optic cannula to an amplifier and laser respectively. After a period of habituation to the recording set-up, stimulation was performed with a diode laser (473 nm; LaserGlow) controlled by Spike 2 software (CED). ECG (gain: 2K, band pass filter: 10–1000 Hz) signal was acquired (sampling rate: 1K) and heart rate calculated using Spike 2 software. Stimulation consisted of a 10 s period of stimulation at 10 Hz (5 ms pulse) with a power output at the tip of the connecting fiber of 12 mW.

To assess the esophageal effects of nAmb stimulation, mice were anaesthetized with ketamine (150 mg/kg) and dexmedetomidine (1 mg/kg). Depth of anesthesia was assessed by absence of the corneal and hind-paw withdrawal reflex. Additional anesthetic was administered as necessary (10% of the original dose, intraperitoneal). Body temperature was maintained at 37.2 ± 0.5°C with a servo-controlled temperature pad (TC-1000; CWE). Following induction of anesthesia, the fiber optic was connected to the laser, and mice were then placed in a stereotaxic frame in the supine position. A midline incision followed by blunt dissection and retraction of overlying tissue was performed to expose the trachea. A tracheostomy was performed, and mice were mechanically ventilated with pure oxygen (MiniVent type 845; Hugo-Sachs Electronik). Mice were ventilated at volume of 150–250 uL and rate between 150 and 250 breaths/min. Mice were hyperventilated to eliminate spontaneous breathing efforts. The trachea was bisected and reflected exposing the anterior surface of the esophagus. Focal stimulation of the exposed surface of the esophagus was conducted using a multimode 200um fiber (0.39NA, Thorlabs) positioned using a micromanipulator. Stimulation was performed with a diode laser (473 nm; LaserGlow) controlled by Spike 2 software (CED). For video imaging of contractions with focal stimulation of the esophagus, 10 ms pulses of laser light (12 mW measured at the tip) was delivered at 2 or 5 Hz for between 5 and 10 s. Video was acquired with a high-speed color CMOS Camera mounted to the operating microscope. Videos were acquired at a framerate of 50–60 Hz. Video scoring was performed by scorers blinded to the genotype of the mice. Scorers were first trained to identify light-evoked contractions and then instructed to count contractions in each video. As the number of light pulses and thus contractions varied across individual videos, scorer accuracy is represented as the number of observed contractions as a percentage of laser flashes for each video.

Contractile force in the esophagus during optogenetic stimulation was assessed during cell-body stimulation of Crhr2^nAmb^ neurons. Mice were anesthetized as described above. Following induction of anesthesia, a fiber optic was connected to an implanted fiber optic cannula, and mice were then placed in a stereotaxic frame in the supine position. Mice were ventilated as described above. To measure contractile force in the esophagus, a miniature latex balloon (Harvard Apparatus) and catheter filled with saline was advanced through the mouth into the upper esophagus. Placement was visually confirmed under an operating microscope. The balloon was connected to a pressure transducer and amplifier and filter (gain: 5K, band pass filter: 0.1–50 Hz, CWE inc.) The balloon was inflated with saline to a pressure between 20 and 50 mmHg during periods of stimulation. Stimulation was performed with a diode laser (473 nm; LaserGlow) controlled by Spike 2 software (CED). Stimulation consisted of a single 1 s pulse or a 10 s of stimulation at 2–10 Hz (5 ms pulse).

### QUANTIFICATION AND STATISTICAL ANALYSIS

No statistical tests were performed except for in the sNuc-Seq analysis, where details are provided above, and the optogenetics heart rate data. Two-way ANOVA was used to compare heart rate changes between genotypes and baseline heart rate compared to stimulation. The group sizes (n) used in each experiment are indicated in the Results.

## Supplementary Material

1

## Figures and Tables

**Table:** KEY RESOURCES TABLE

REAGENT or RESOURCE	SOURCE	IDENTIFIER
Antibodies		
Rabbit anti-PLAP	Abcam	Cat # ab133602; Lot # GR323330-4
Goat anti-Chat	Sigma Aldrich	Cat # AB144P; Lot # 3491643
Chicken anti-GFP	Aves Lab	Cat # GFP-1020; Lot GFP3717982
Donkey anti-rabbit	Invitrogen	Ref: A31573; Lot: 2083195
Donkey anti-goat 488	Invitrogen	Ref: A11055; Lot: 1942238
Donkey anti-goat 647	Invitrogen	Ref: A21447; Lot: 2273668
Donkey anti-Chicken 488	Jackson ImmunoResearch Laboratories	Code: 703-545-155; Lot: 144438
Bacterial and virus strains		
AAVDJ-DIO-H2b-mCherry	Tao et al. (2021)	N/A
AAV9-CAG-FLEX-PLAP	Prescott et al., 2020	N/A
Chemicals, peptides, and recombinant proteins		
Fluorogold	Fluorochrome	Cat: Fluoro-gold; RRID: AB_2314408
TSA Plus Fluorescein Reagent	Akoya Biosciences	Cat #: TS-000200
TSA Plus Cy3 Reagent	Akoya Biosciences	Cat #: TS-000202
TSA Plus Cy5 Reagent	Akoya Biosciences	Cat #: TS-000203
Critical commercial assays		
RNAscope Multiplex Fluorescent Reagent Kit V2	Advanced Cell Diagnostics	Cat #: 323100
NexteraXT DNA Library Preparation Kit	Illumina	Cat #: FC-131-1096
Nextera XT Index Kit v2 Set A	Illumina	Cat #: FC-131-2001
Nextera XT Index Kit v2 Set B	Illumina	Cat #: FC-131-2002
Nextera XT Index Kit v2 Set C	Illumina	Cat #: FC-131-2003
Nextera XT Index Kit v2 Set D	Illumina	Cat #: FC-131-2004
Deposited data		
Raw and processed single-nuclei RNA-seq data files	GEO	GSE202760
Clustered single-nuclei RNA-seq data	Broad Single Cell Portal	https://singlecell.broadinstitute.org/single_cell/study/SCP1677
Experimental models: Organisms/strains		
Mouse/Chat-IRES-Cre	Jackson Laboratory; Rossi et al. (2011)	Cat #: 031661; RRID: IMSR_JAX:031661
Mouse/Phox2b-Flp	Jackson Laboratory; Hirsch et al. (2013)	Cat #: 022407; RRID: IMSR_JAX:022407
Mouse/Crhr2-CreEGFP	Anthony et al., 2014	N/A
Mouse/Vipr2-IRES-Cre	Jackson Laboratory; Daigle et al. (2018)	Cat # 031332; RRID:IMSR_JAX:031332
Mouse/Chat-p2a-Flp	This paper	N/A
Mouse/CaTCh	Jackson Laboratory; Daigle et al. (2018)	Cat # 025109; RRID:IMSR_JAX:025109
Oligonucleotides		
Mm-Chat	Advanced Cell Diagnostics	Cat #: 408731
Mm-Chat-C2	Advanced Cell Diagnostics	Cat #: 408731-C2
Mm-Chat-C3	Advanced Cell Diagnostics	Cat #: 410071-C3
Mm-Adcyap1	Advanced Cell Diagnostics	Cat #: 405911
Mm-Adcyap1-C2	Advanced Cell Diagnostics	Cat #: 405911-C2
Mm-Crhr2-C2	Advanced Cell Diagnostics	Cat #: 413201-C2
Mm-Crhr2-C3	Advanced Cell Diagnostics	Cat #: 413201-C3
Mm-Vipr2	Advanced Cell Diagnostics	Cat #: 465391
Mm-Vipr2-C2	Advanced Cell Diagnostics	Cat #: 465391-C2
Mm-CRE-C3	Advanced Cell Diagnostics	Cat #: 312281-C3
Mm-Flp	Advanced Cell Diagnostics	Cat #: 1157161-C1
Mm-Phox2b-C2	Advanced Cell Diagnostics	Cat #: 407861-C2
Mm-Phox2b-C3	Advanced Cell Diagnostics	Cat #: 407861-C3
Mm-Calca-C2	Advanced Cell Diagnostics	Cat #: 417961-C2
Mm-Calca-C3	Advanced Cell Diagnostics	Cat #: 417961-C3
Software and algorithms		
R	R version 3.6	https://www.r-project.org/; RRID: SCR_001905
Seurat v4.0	Stuart et al., 2019	https://github.com/satijalab/seurat/; RRID: SCR_007322
Seurat code used for cell clustering	Zenodo	https://doi.org/10.5281/zenodo.6564316
Illustrator	Adobe	https://www.adobe.com; RRID: SCR_010279
Excel	Microsoft	https://www.microsoft.com/en-us/; RRID: SCR_016137
bcl2fastq v2.20.0	Illumina	https://support.illumina.com/sequencing/sequencing_software/bcl2fastq-conversion-software.html; RRID: SCR_015058
STAR v2.6.1	Dobin et al., 2013	https://github.com/alexdobin/STAR

## Highlights

- The nucleus ambiguus (nAmb) comprises at least three molecular subtypes of neurons
- Vipr2^+^ nAmb neurons are mostly in the caudal nAmb and innervate pharynx, larynx
- Crhr2^+^ nAmb neurons are mostly in the rostral nAmb and innervate esophagus
- Activating Crhr2^+^ nAmb neurons contracts esophagus but does not affect heart rate
