# Supplementary material for: Genetic encoding of an esophageal motor circuit
Source: Cell Rep. Author manuscript; Available in PMC 2022 Jul 5. (PMC9255432; doi:10.1016/j.celrep.2022.110962)
Supplement: 1 [file NIHMS1816596-supplement-1.pdf]

Cell Reports, Volume *39*

Supplemental information

Genetic encoding of an esophageal motor circuit

Tatiana C. Coverdell, Ruei-Jen Abraham-Fan, Chen Wu, Stephen B.G. Abbott, and John N. Campbell

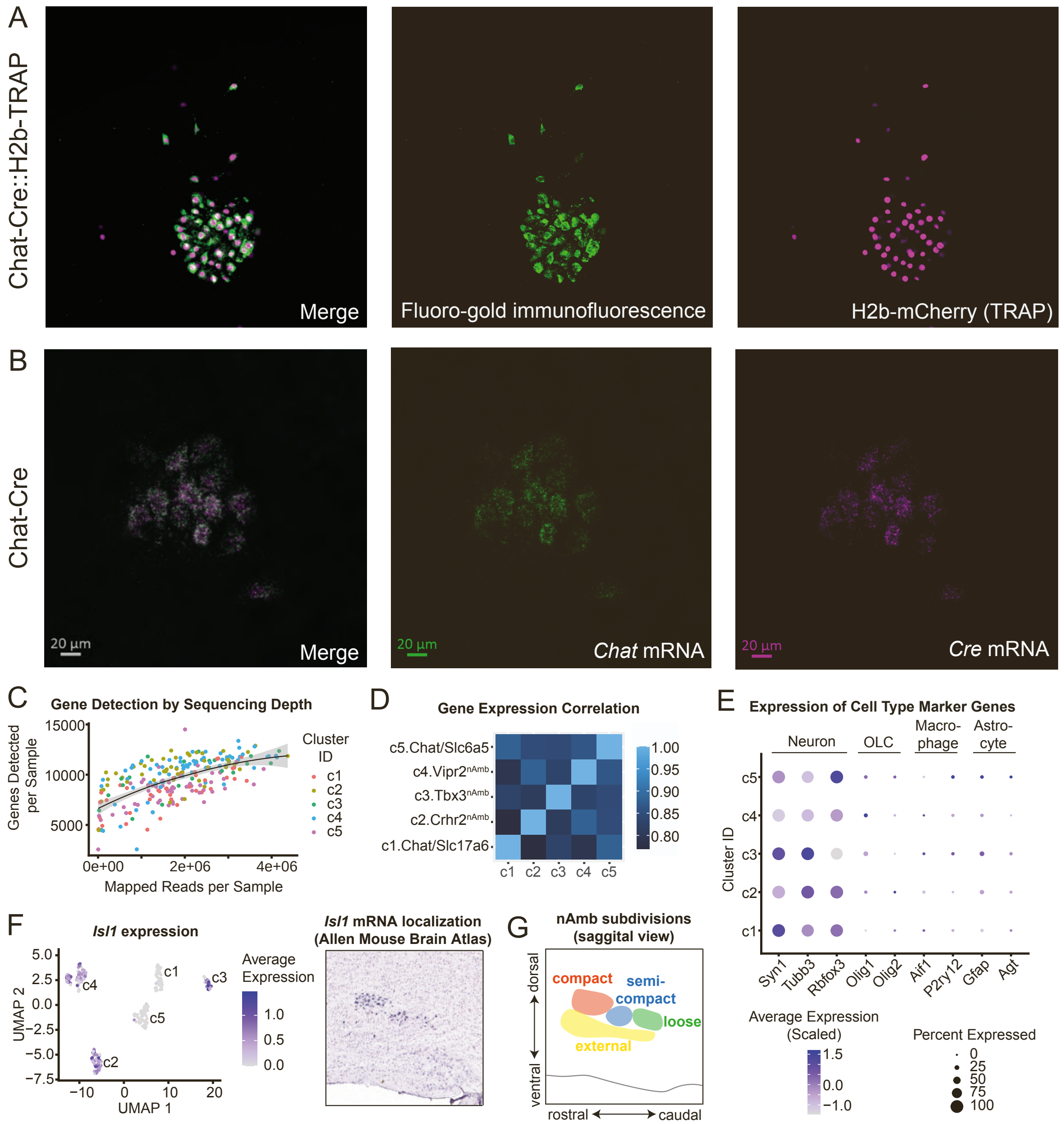

Fig. S1. Validation of Chat-Cre Activity and Anatomical and Cellular Identity. Related to Figure 1.

A. Co-localization of Fluoro-gold and H2b-mCherry in the nAmb of Chat-Cre::H2b-TRAP mice (n=3 mice).

B. Co-localization of *Cre* mRNA and *Chat* mRNA in the nAmb of Chat-Cre mouse by RNA fluorescence *in situ* hybridization (RNA FISH; image representative of 3 mice; 103 of 103 *Chat*+ neurons were also *Cre*+; no *Cre* mRNA detected in hindbrain cells that did not express *Chat*).

C. Relationship of sequencing depth to gene detection in individual sNuc-seq samples (dots, colored by cluster identity)

D. Pairwise correlation of gene expression profiles between each pair of cell clusters

E. Cluster-level expression of neuron, oligodendrocyte lineage cells (OLC), macrophage, and astrocyte marker genes

F. Left, log-normalized expression of the nAmb marker gene, *Isl1*, superimposed on UMAP. Right, *in situ* hybridization (ISH) of *Isl1* mRNA in a sagittal section of the nAmb (Allen Mouse Brain Atlas, experiment 597) (Lein et al., 2007).

G. Map of nAmb subregions based on *Isl1* ISH data in Fig. S1F.

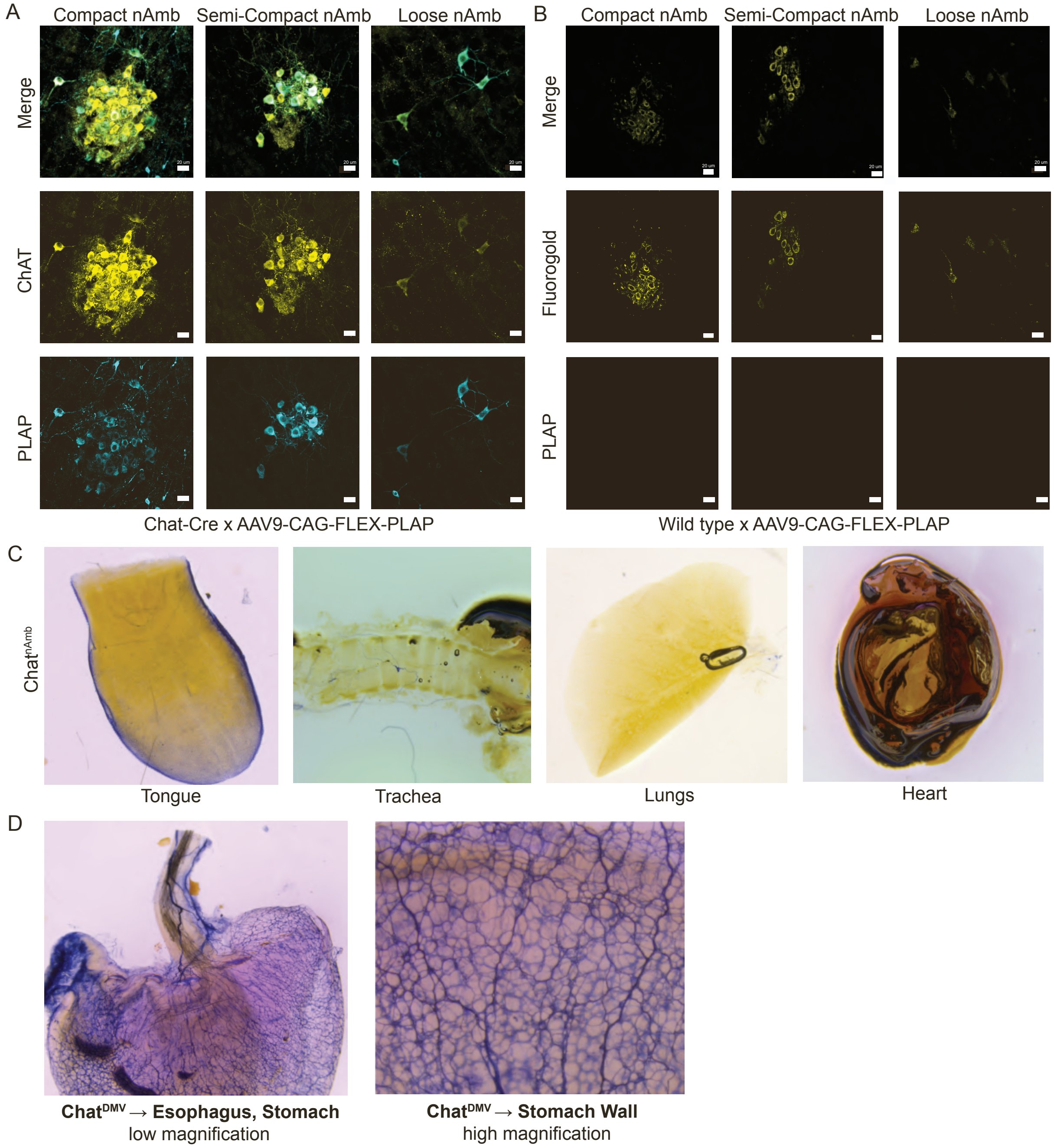

Fig. S2. Immunofluorescence Images of AAV9-CAG-FLEX-PLAP Expression in Chat-Cre and Wild type Mouse Lines Confirm Successful Infection and Expression; Chat^{nAmb} Innervation of the Tongue, Trachea, Lungs, and Heart; Chat^{DMV} Innervation of the Esophagus and Stomach. Related to Figure 3.

A. Colocalization of ChAT and PLAP immunofluorescence in nAmb neurons after injection of AAV9-CAG-FLEX-PLAP into the nAmb of a Chat-Cre mouse (n=6 mice).
B. Colocalization of ChAT and PLAP immunofluorescence in nAmb neurons after injection of AAV9-CAG-FLEX-PLAP into the nAmb of a wildtype mouse (n=3 mice).
C. PLAP stained axons in the tongue, trachea, lungs, and heart of Chat-Cre mice following injection of AAV9-CAG-FLEX-PLAP into the nAmb. (n=6 mice)
D. PLAP stained axons in the esophagus and stomach of Chat-Cre mice following injection of AAV9-CAG-FLEX-PLAP into the DMV (n=3 mice). Higher magnification image of PLAP stained axons in the stomach wall of Chat-Cre mice following injection of AAV9-CAG-FLEX-PLAP into the DMV (n=3 mice).

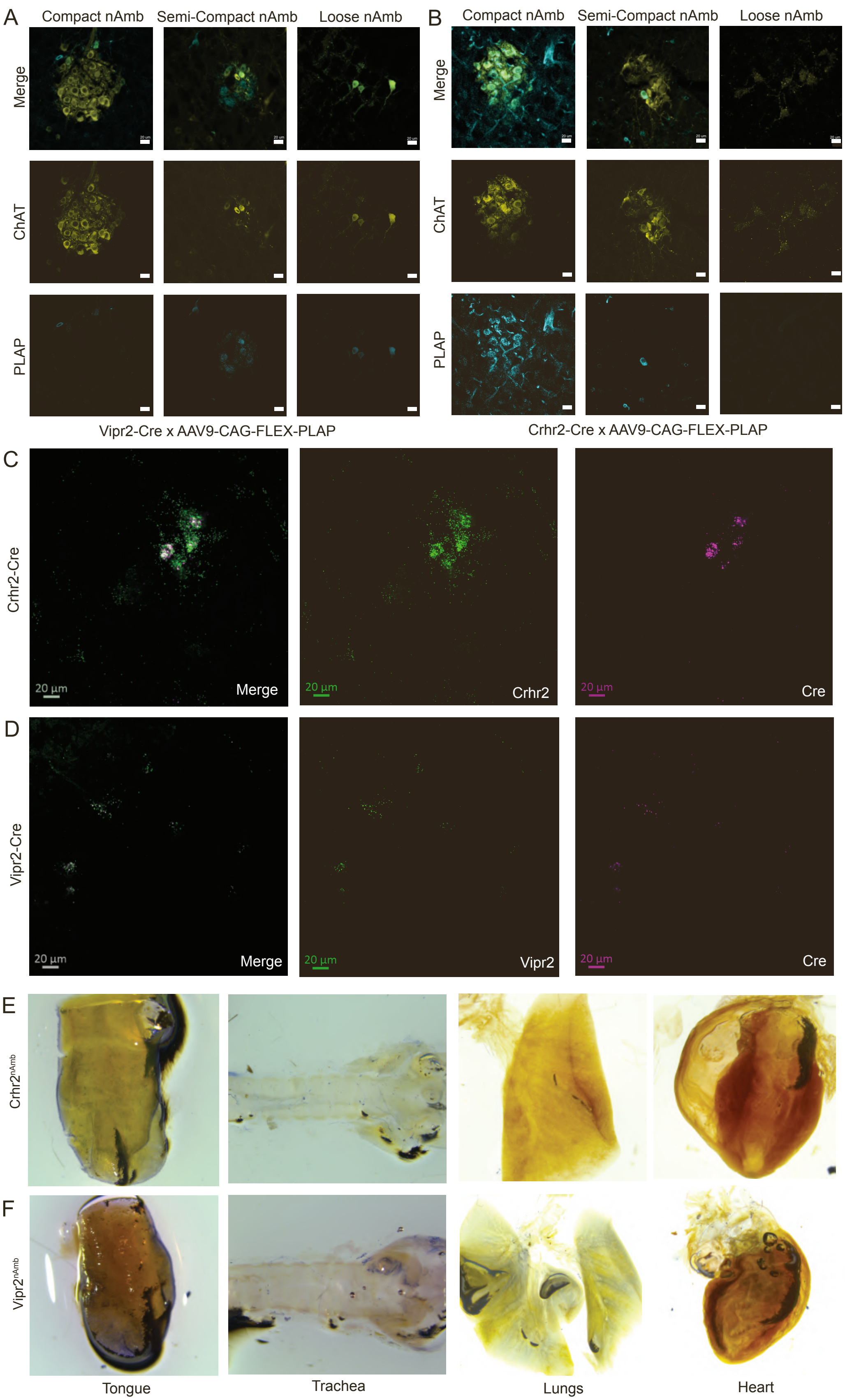
A
Compact nAmb
Semi-Compact nAmb
Loose nAmb
Merge
ChAT
PLAP
Vipr2-Cre x AAV9-CAG-FLEX-PLAP
B
Compact nAmb
Semi-Compact nAmb
Loose nAmb
Merge
ChAT
PLAP
Crhr2-Cre x AAV9-CAG-FLEX-PLAP
C
Crhr2-Cre
20 µm
Merge
20 µm
Crhr2
20 µm
Cre
D
Vipr2-Cre
20 µm
Merge
20 µm
Vipr2
20 µm
Cre
E
Crhr2nAmb
F
Vipr2nAmb
Tongue
Trachea
Lungs
Heart

Fig. S3. Immunofluorescence Images of AAV9-CAG-FLEX-PLAP Expression in Vipr2-Cre and Crhr2-Cre Mouse Lines Validate Infection and Expression; Validation of Crhr2-Cre and Vipr2-Cre Activity; Vipr2^{nAmb} and Crhr2^{nAmb} Innervation of the Tongue, Trachea, Lungs, and Heart. Related to Figure 3.

A. Colocalization of ChAT and PLAP immunofluorescence in nAmb neurons after injection of AAV9-CAG-FLEX-PLAP into the nAmb of a Vipr2-Cre mouse (n=5 mice).
B. Colocalization of ChAT and PLAP immunofluorescence in nAmb neurons after injection of AAV9-CAG-FLEX-PLAP into the nAmb of a Crhr2-Cre mouse (n=5 mice).
C. Co-localization of *Cre* mRNA and *Crhr2* mRNA in the nAmb of Crhr2-Cre mouse by RNA fluorescence *in situ* hybridization (RNA FISH; image representative of 4 mice; 79 of 82, or 96%, *Crhr2*+ neurons were also *Cre*+; no *Cre* mRNA detected in hindbrain cells that did not express *Crhr2*).
D. Co-localization of *Cre* mRNA and *Vipr2* mRNA in the nAmb of Vipr2-Cre mouse by RNA fluorescence *in situ* hybridization (RNA FISH; image representative of 3 mice; 57 of 57 *Vipr2*+ neurons were also *Cre*+; no *Cre* mRNA detected in hindbrain cells that did not express *Vipr2).*
E. Little to no PLAP stained axons in the tongue, trachea, lungs, and heart of Crhr2-Cre mice following injection of AAV9-CAG-FLEX-PLAP into the nAmb (n=5 mice).
F. Little to no PLAP stained axons in the tongue, trachea, lungs, and heart of Vipr2-Cre mice following injection of AAV9-CAG-FLEX-PLAP into the nAmb (n=5 mice).

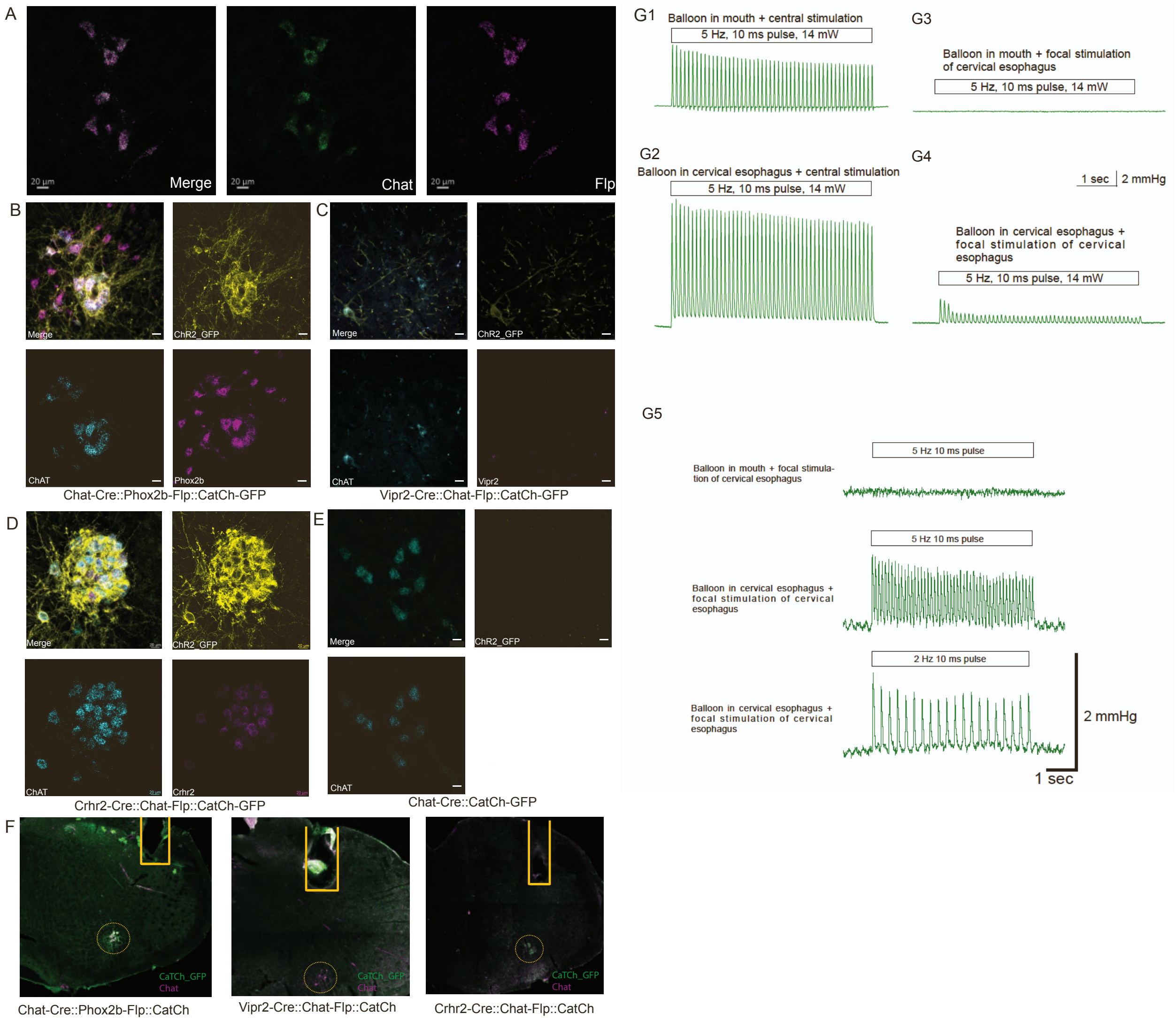

Fig. S4. Validation of Chat-Flp Expression; Validation of Subtype Specific Cre::Flp::CatCh Mouse Lines and Fiber Implant Locations; Stimulation of Cholinergic *Crhr2*+ Neurons in the Brain and Esophagus Contracts the Cervical Esophagus. Related to Figure 4.

A. Co-localization of *Flp* mRNA and *Chat* mRNA in the nAmb of Chat-Flp mouse by RNA fluorescence *in situ* hybridization (RNA FISH; image representative of 3 mice; 121 of 121 *Chat*+ neurons were also *Flp*+; no *Flp* mRNA detected in hindbrain cells that did not express *Chat*.
B. Co-localization of *Chat* RNA FISH, *Phox2b* RNA FISH, and eYFP immunofluorescence in Chat-Cre::Phox2b-Flp::CaTCh-eYFP mouse (n=3 mice).
C. Colocalization of *Vipr2* RNA FISH, *Chat* RNA FISH, and eYFP immunofluorescence in Vipr2-Cre::Chat-Flp::CaTCh-eYFP mouse (n=3 mice).
D. Colocalization of *Crhr2* RNA FISH, *Chat* RNA FISH, and eYFP immunofluorescence in Crhr2-Cre::Chat-Flp::CaTCh-eYFP mouse (n=3 mice).
E. Colocalization of *Chat* RNA FISH and eYFP immunofluorescence in Cre::CaTCh-eYFP mouse. (n=3 mice)
F. Representative images of optic fiber tracts, eYFP and ChAT immunofluorescence from Chat-Cre::Phox2b-Flp::CaTCh-eYFP, Vipr2-Cre::Chat-Flp::CaTCh-eYFP, and Crhr2-Cre::Chat-Flp::CaTCh-eYFP mice used for optogenetic experiments
G. Changes in pressure measured in the mouth and cervical esophagus generated by central and focal stimulation in Crhr2-Cre::Chat-Flp::CaTCh mice. Central stimulation (G1, G2) produced pressure deflections whether the balloon was positioned in the mouth (G1) or cervical esophagus (G2), however the peak amplitude of pressure deviations was the greatest when measured in the esophagus. Focal application of light on the cervical

esophagus (G3, G4) produced pressure deviations when the balloon was positioned in the esophagus (G4), but not in the mouth (G3). Focal application of light on the cervical esophagus produces frequency-dependent increases in pressure deviations (G5).
